# The Provision for Nursing Fevers in London

**Published:** 1886-11-27

**Authors:** 


### THE PROVISION FOR NURSING FEVERS IN LONDON.

Importance of the Subject.

The nursing of persons suffering from acute infectious disease is now properly regarded as the more important part of their medical treatment. Physicians recognise the impossibility of cutting short maladies of this nature. All such illnesses run their course, and the aim of those who have charge of the sufferers is to enable them to live beyond the time when the fever takes its departure. Hence the treatment of fever and small-pox is more or less limited to the placing of the patient under proper hygienic conditions, to providing him with appropriate food, and to giving him all the opportunity for rest and quiet, which may serve to maintain his powers, often sorely taxed by the consuming malady to which he is subjected. It is evident that the influence of the nurse is for these purposes not less important than that of the physician, whose duty often consists in merely directing the former in her care of her patient. It is no exaggeration to state that special training, both for nurse and doctor, is essential if the patient is to have the best chances of recovery. Lives have too often been lost by the placing of the sufferer in an inappropriate position, or by the administration of food not suited to his condition. The unceasing watchfulness, the care in detail which are required in the management of a case of infectious disease are often known only to those who have been associated with large public institutions; and the employment of nurses whose training does not include the acquirement of experience of this sort, is a very palpable danger to which the fever-stricken poor are exposed.

Non-Epidemic Periods.

It is doubtless these considerations which have led the Metropolitan Asylums Board to report upon the question of retaining the services of such of their officers as may not be required during non-epidemic periods. Every epidemic in the metropolis brings into relation with the sick who enter the hospitals of the Metropolitan Asylums Board a large number of women who in no way can claim to have the knowledge and experience we hold to be necessary for the proper performance of their duties to their patients; and the close of every epidemic leads to their dispersion, and makes it necessary for another and an untrained staff to be brought together the next time epidemic disease assumes excessive prevalence. Many women thus discharged from their employment at the close of an epidemic find it impossible to obtain work as nurses, and are obliged to seek another occupation, so that their services are not available for the next outbreak of infectious disease. At the present moment as many as three of the six fever hospitals under the control of the managers are not required for the treatment of fever patients, and are consequently closed, their nursing staff have been discharged, and nurses of excellent character, who have served the Board faithfully and well for periods ranging from five to fifteen years, have been compelled to accept inferior positions at others of the Board's hospitals, or to seek a livelihood elsewhere. At the same time, fever is spreading in the metropolis; it may not be long before the now empty hospitals are again filled with patients, who will be largely dependent upon an untrained staff for their chances of recovery.

The Evils of a Bad System.

The unfortunate results which are attendant upon this policy are, therefore, mischievous in two senses; this hand-to-mouth method of supplying the requirements of London hospitals necessitates on the one hand an act of grave injustice to the nurses themselves, in compelling them to lose the benefits which would accrue to those who do their work honestly in other public service which they enter, but it also tends to hinder many who might render important services to the metropolis from engaging in an occupation of so uncertain a character. The chief sufferers are, however, the patients themselves, who enter the hospitals before the newly-appointed staff are able to bring skill and experience to bear upon their work. Nor must be forgotten the unfair responsibility which is thus cast upon the medical staff in their endeavours to do justice to the patients who come under their care. If necessary, objections could be multiplied against this system, for every epidemic adds to the list of nurses who themselves contract infectious disease from their patients, and while many a useful life is lost in the struggle, others are rendered safe from further risk. The loss of life, the loss of money, expended during the illness of the nurse, makes it urgent that some other course should be adopted than that which is attended by these serious disadvantages.

The Remedy.

But is there a remedy? It is obvious that the retention of nurses in the employment of the Metropolitan Asylums Board for many months when there is no work to be done, would not be of advantage to the ratepayers or to those who are thus kept in enforced idleness. At the end of every epidemic there can always be a useful review of the efficiency of the different nurses in each institution, and while for some it may be said that their sphere is not in hospital work, for others it may be held that the retention of their services would be beneficial to the Metropolis. The question then arises whether this nucleus of a future nursing staff can be usefully occupied to an extent sufficient to prevent the demoralisation which is often the outcome of enforced idleness. For some of these such employment of time could be certainly found, the higher training which is now regarded as necessary for the acquisition of a full knowledge of nursing may readily make claims upon the energies of the more active-minded and industrious of the staff. It would probably not be difficult to make arrangements with the governing bodies of other institutions for the employment of these women, while the continuance of the payment of some portion of their salaries by the Managers would entitle the Metropolitan Asylums Board to a claim upon their services, should occasion arise.

**Nurses on Half-Pay.**

An introduction of something akin to a half-pay system, resembling perhaps the arrangements which are made in military services, may well be the outcome of the consideration of this subject by the committee which has just been appointed. It is possible that some increase of cost would be incurred by the execution of this project, but it must be remembered that it would be money well expended on behalf of the suffering poor. The services which would thus be secured would be of greater monetary value than those which are now available, and if it were possible to extend the principle so far as to include the provision in old age for those who have passed a long life in the service of their fellows, the greatest amount of good would be conferred upon all who, as patients or nurses, come under the control of the Metropolitan Asylums Board.

